# The Potential Cutaneous Effects of Pornography Addiction: A Narrative Review

**DOI:** 10.7759/cureus.33066

**Published:** 2022-12-28

**Authors:** Taha F Rasul, Kayla R Schwartz, Farhan Qureshi, Emily Eachus, Armen Henderson

**Affiliations:** 1 Dr. Phillip Frost Department of Dermatology and Cutaneous Surgery, University of Miami Miller School of Medicine, Miami, USA; 2 Department of Urology, University of Miami Miller School of Medicine, Miami, USA; 3 Department of Internal Medicine, University of Miami Health System, Miami, USA

**Keywords:** labiaplasty, purpura and strangulation marks, excessive masturbation, hypersexual disorder, addictive behavior, sexual addiction, skin conditions, pornography addiction, cutaneous manifestations

## Abstract

Pornography addiction is an area of increasing concern, particularly due to the ubiquitous nature of pornographic material on the Internet. Even so, there is no formal Diagnostic and Statistical Manual of Mental Disorders (DSM-5) inclusion of compulsive pornography use as a behavioral addiction. Although the psychosocial impacts of pornography addiction have been studied, the risk of direct skin injuries and behavioral changes brought about by excessive pornography usage remain to be seen. Adult males constitute the majority of cases of engaging in risky and violent sexual behaviors with an unclear association with pornography consumption. Adult females may be likely to copy pubic hair trimming patterns analogous to those seen in pornographic content, even though attitudes towards labiaplasty are unclear. Finally, adolescents regularly exposed to pornography have been found to replicate sexual activity seen in pornographic material and have earlier sexual activity. In the literature evaluated, an association between pornographic material and direct cutaneous disease remains a major area of further research.

## Introduction and background

The cardinal feature of addictive behaviors is the inability to resist a drive, impulse, or temptation that may be harmful to oneself or others [1]. The majority of addictive behaviors categorized in the Diagnostic and Statistical Manual of Mental Disorders (DSM-5) are substance-related, with only gambling listed in the ‘non-substance-related disorders’ category. While pornography addiction is not categorized as an addictive behavior in the DSM-5, other disorders related to excessive internet use are. For example, internet gaming disorder is listed in the DSM-5 as a subject of further study [2].

Pornography addiction can be facilitated through non-digital erotica like magazines and books, but the accessibility, affordability, and anonymity afforded by internet-based materials have made online pornography access and usage ubiquitous, especially during the coronavirus disease 2019 (COVID-19) pandemic [3]. Still, there is currently a lack of empirical data on behaviors like excessive masturbation, telephone sex, and pornography use that fall under the ‘hypersexual disorder’ umbrella [4]. 

Hypersexual disorders generally manifest with overwhelming sexual urges, fantasies, and behaviors leading to clinically significant psychological impairment. Cutaneous manifestations of such disorders, including pornography addiction, have not been studied extensively [5]. It is plausible that overwhelming sexual urges, leading to excessive masturbation, may have direct effects on hair, skin, or nails. There is limited data on comorbid relationships between addictive and cutaneous disorders with two notable exceptions: (i) trichotillomania and skin-picking disorder, and (ii) substance addiction and gambling disorder, the latter of which has been shown to have a genetic component [6].

Cutaneous manifestations of pornography addiction are important for both patients and practitioners because of the potential impact on overall health. Currently, there is a critical knowledge gap regarding pornography addiction and its effects on the skin. This review assessed the genitocutaneous and sociobehavioral effects of pornography addiction and patterns among adults and adolescents.

## Review

Methods

Overview

A literature search was conducted on PubMed, Embase, and Cochrane databases for the concepts of “masturbation”, “pornography”, “skin injury”, synonymous terms, and medical subject headings (MeSH) terms. A combination of database subject terms and keywords, such as Emtree, were also utilized. Studies included case reports, systematic reviews, observational studies, and controlled trials. The article was prepared using the Scale for the Assessment of Narrative Review Articles (SANRA) [7].

Search Strategy and Information Sources

The search for literature was performed and included articles published from January 1, 1970, to January 1, 2022. The following search terms were utilized for each database:

PubMed: ((masturbation) OR (porn* or pornography or erotica)) AND ("Skin Diseases"[Mesh])

EMBASE PICO: ('pornography'/exp OR 'pornography') AND ('skin'/exp OR 'skin'); ('pornography'/exp OR 'pornography') AND ('masturbation'/exp OR 'masturbation') AND ('skin'/exp OR 'skin'); ('masturbation'/exp OR 'masturbation') AND ('skin'/exp OR 'skin')

Cochrane: MeSH descriptor: #1 [Masturbation] explode all trees; #2 pornography, #3 skin, #4 #2 AND #3

Inclusion and Exclusion Criteria

Case series, case reports, and original research, which mentioned cutaneous injuries from masturbation with or without pornographic association, were included. Inclusion criteria were studies: (i) detailing dermatologic diseases associated with excessive masturbation, (ii) describing the relationship between excessive masturbation and pornography usage, and (iii) dermatologic associations with pornography addiction. On the other hand, articles that did not directly address the cutaneous (or extracutaneous) effects of excessive masturbation or pornography addiction were excluded. More specifically, articles that lacked information about the relationship between cutaneous injury and excessive masturbation or cutaneous injury and pornography addiction met exclusion criteria.

Article Selection and Analysis

**Figure 1:**
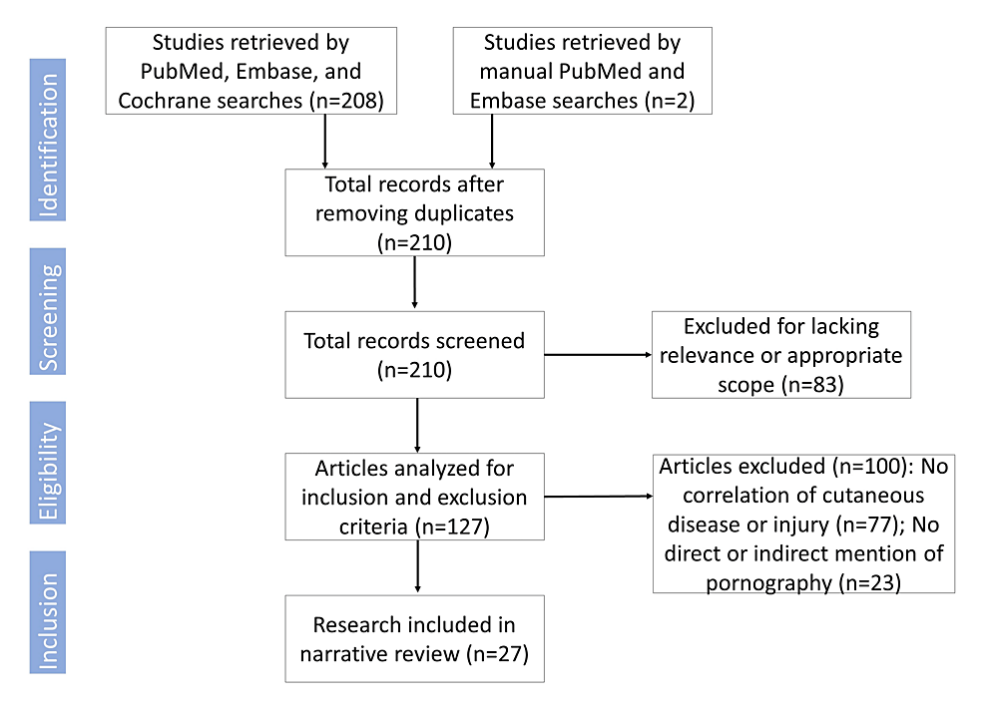
Flowchart describing article identification, screening, eligibility, and inclusion.

Among 208 articles isolated, 27 met the inclusion criteria and were included in this synthesis (Figure 1). A further three articles were retrieved through manual PubMed and Embase searches. The studies retrieved and evaluated can be found in the Appendix.

Sociobehavioral or genitocutaneous changes with and without pornographic associations

Fourteen studies included in this review, which show some connection between masturbation, pornography, sociobehavioral changes, or skin injuries, are described in Table 1.

**Table 1:** Studies describing masturbation or pornography with cutaneous or extracutaneous effects and further divided by adult males, adult females, and adolescents. AAD: accidental death in autoerotic maneuvers; OR: odds ratio; CI: confidence interval; STD: sexually transmitted disease; STI: sexually transmitted infection; HIV: human immunodeficiency virus

	Type of study	Initial presentation/Premise of study	Cutaneous manifestations	Association with pornography or erotica	Extra-cutaneous manifestations	Treatment or additional information
Adult Males						
Verma et al. 2020 [8]	Case Report	23-year-old male presented with a painless blister following masturbation the same morning.	Hemorrhagic bulla superimposed on penile lichen planus.	Unclear. Not explicitly mentioned.	Localized to glans and prepuce only.	Treated with aspiration and topical 0.1% tacrolimus
Ghiya et al. 2008 [9]	Case report	30-year-old male presented with a 3-month history recurrent itching, swelling, and redness a few minutes after masturbation that persisted for 4-6 hours.	Masturbation-induced urticaria. Symptoms did not occur during protected or unprotected vaginal intercourse. No other history of dermographism or urticaria.	Unclear. Not explicitly mentioned.	Localized to penis.	Treated with hydroxyzine 10 mg and prednisolone 20 mg for 5 days which prevented further urticaria.
Heiner et al. 2012 [10]	Case report	29-year-old male with a history of masturbation-induced penile erythema presented with a two-day history of fever, severe scrotal pain, and diffuse myalgias. He endorsed frequent masturbation using soap as a lubricant.	Significant erythema, edema, and warmth of area including penis (but sparing glans), scrotum, and pubic symphysis. Large eschar noted on ventral penis. Fournier’s gangrene diagnosed.	Unclear. Not explicitly mentioned.	No gangrenous extension to bladder or rectum. Patient suffered from septic shock with a complicated hospital course.	Multiple debridement surgeries, aggressive antibiotic treatment, and split-thickness skin grafting.
Focardi et al. 2018 [11]	Case series	Report of three cases. Case 1, 52-year-old man partially hung by neck and ankles to ceiling. Case 2, 31-year-old man found on bed with canisters of butane gas. Case 3, 52-year-old man found on couch with multiple medications (including sildenafil and diazepam) and used syringes.	Accidental death in autoerotic maneuvers (AAD). Case 1, soft furrow of skin in neck demonstrated likely cardio-respiratory arrest secondary to mechanical asphyxia. Case 2, numerous petechiae and purpura. Case 3, puncture wounds.	Pornographic materials (pictures, magazines) found in each case. Active masturbation near the time of death was occurring in Case 2 and Case 3.	Case 2: poly-visceral congestion likely due to acute asphyxia.	All deaths were ruled accidental. None of the patients had prior psychiatric diagnoses. Epidemiological data shows high prevalence of men in AAD.
Vennemann et al. 2006 [12]	Case report	30-year-old man found hanged in living room. Accidental death in autoerotic maneuver. Left hand was in trousers in genital region.	Strangulation marks on bilateral neck. Also had findings of advanced sarcoidosis, including nasal granulomas.	Violent pornographic content found on computer which was likely being viewed close to time of death.	Iridocyclitis, uveitis, and diffuse granulomas in bilateral lungs, spleen, and abdominal lymph nodes.	Suicide could have been provoked by advanced sarcoidosis, which had poor prognosis due to nasal granulomas and chronic uveitis.
Kyomukama et al. 2021 [13]	Case report	43-year-old male presented with a 72-hour-history of a ball bearing wrapped around penile shaft.	Ulcerated areas on penis, grossly edematous penile shaft with serosanguinous discharge.	Patient had a history of pornography addiction.	Primarily genitourinary involvement.	Surgical debridement and broad spectrum antibiotics.
Adult Females						
Goldmeier et al. 2008 [14]	Case series	Report of six cases of women with persistent genital arousal disorder and evaluation of different parameters of the disorder including cutaneous, psychosocial, and physiological.	Persistent genital arousal disorder coexisted with dermatoses in certain cases.	Unclear. Not explicitly mentioned. Masturbation is often used as a method to reduce feelings of genital arousal.	Primarily engorgement of genitalia.	Associated with neurophysiological conditions such as anxiety and pathologic conditions like genitopelvic prolapse.
Schick et al. 2010 [15]	Cross-sectional study	217 undergraduate women surveyed for satisfaction of genital appearance and sexual activity.	General dissatisfaction with genital appearance (labia majora, labia minora, pubic hair, etc.) was associated with decreased sexual satisfaction and decreased likelihood of pursuing risky sexual behaviors that could be injurious to genitalia.	Increased exposure to pornography may increase feelings of distress about genital appearance.	Self-esteem and self-consciousness issues primarily.	Sample was primarily Caucasian, heterosexualcollege-aged women.
Dubinskaya et al. 2022 [16]	Cross-sectional study	25 most-viewed videos from popular pornography sites were reviewed. Female genital appearance, hair grooming, pigmentation, and labial dimensions were assessed.	Among male and female performers, complete pubic hair shaving was the most common pattern, followed by trimming. 8% of videos showed surgically enhanced labia majora. Most videos had a range of genital appearance, weakening association with labiaplasty, but reinforcing a connection with hair grooming patterns.	Explicitly pornographic videos from the five most popular free pornography websites.	No other pelvic organ prolapse signs, piercings, or extracutaneous findings noted.	
Mowat et al. 2015 [17]	Systematic review	Online pornographic content and female attitudes towards genital cosmetic surgery.	Vulvar diversity pathologized in most of cyberspace, “clean slit” vulva perceived as ideal.	Internet and pornography may have a role in women’s likelihood of undergoing labiaplasty surgery.	Primarily psychological manifestations including self-esteem.	Lack of definitive data on association between pornography and labiaplasty.
Children/Adolescents						
Kinjo et al. 2019 [18]	Case report	14-year-old boy with fever, urinary incontinence, and scrotal pain. Physical examination showed induration at pubic symphysis.	Urethrocutaneous fistula and abscess secondary to spherical magnets inserted into urethra one year prior to facilitate masturbation.	Unclear. Not explicitly mentioned.	Fever, urethrocutaneous fistula.	According to a review of Japanese literature, among 51 cases of urethrovesical foreign body in patients under 18, masturbation was the cause of 31 (73.8%).
Thompson et al. 2016 [19]	Case report	14-year-old boy presented to the emergency department with dyspnea, chest pain, lightheadedness, and fatigue.	Salicylate toxicity. Used 60-gram tube of methylsalicylate cream as lubricant to masturbate. No overt cutaneous lesions.	Unclear. Not explicitly mentioned.	High anion gap metabolic acidosis.	Absorption was likely at scrotal skin, which has been shown to have a 40-fold greater absorption of certain compounds compared to other dermal areas.
Wong et al. 2009 [20]	Case-control	500 Singaporean adolescents assessed for sexual activity predictors.	No overt cutaneous manifestations described.	Among boys, risk factors for early sexual activity included pornography exposure [OR]: 5.82 [95% confidence interval [CI]: 2.34-14.43].		
Ng et al. 2016 [21]	Cross-sectional	300 male adolescents aged 16-19 at an STD clinic in Singapore, assessing for predictors of sexual activity.	Diagnosed STI’s after sexual encounters with female sex workers. These include genital herpes, genital warts, molluscum contagiosum, infectious syphilis, pubic lice, and HIV.	Increased frequency of pornography viewing was associated with more encounters with female sex worker (adjusted prevalence ratio 1.47, CI: 1.04-2.09).	STI involvement including urethritis, pharyngitis, proctitis	

High-risk autoerotic maneuvers and genital injuries in males

In rare cases, adult males engaging in excessive masturbation had direct skin manifestations like hemorrhagic bullae, urticaria, or even Fournier’s gangrene [8-10]. Additionally, males were more likely to engage in risky behaviors like autoerotic asphyxiation, often leading to unintentional death [11,12]. These cases had some form of pornographic materials during the event such as magazines, webpages, and pictures. Overt skin findings include purpura and strangulation marks. Penile strangulation is also an uncommon phenomenon whereby constricting objects (usually metal) are placed around the penile shaft to increase erotic arousal. These cases are usually emergencies and present with overt penile injury including edema and necrosis [13]. There have been only a few overt cases of penile strangulation having pornography as a patient risk factor [13]. 

For adult males, the increased likelihood of engaging in dangerous autoerotic maneuvers may have underlying biopsychosocial causes [22]. Even though there is similar behavior reported in women, the majority of practitioners are men. Therefore, the tendency to partake in such maneuvers may not solely be attributed to erotic material or pornography addiction. However, engaging in such maneuvers has a high risk of cutaneous injury and even death as evidenced by the numerous case reports of accidental death during autoerotic asphyxiation. In a review of autoerotic deaths in the literature from 1954-2004, it was found that most victims were Caucasian males (390/408 cases), with no evaluation of pornographic materials used [23]. This represents a potential area of future research as only one case report demonstrated accidental death while ostensibly trying to replicate violent pornographic material viewed on the internet [12]. 

Similarly, in the cases of masturbation-induced urticaria and hemorrhagic bullae with lichen planus, the association with pornographic or erotic materials was not clearly mentioned [8,9]. There may be hesitation to mention this during physician encounters, especially if lesions on the genitalia are already uncomfortable for patients. Providers should consider including pornography consumption as a potential screening question for patients with atypical genital lesions. 

Attitudes towards pubic hair trimming or labiaplasty in women after exposure to pornography

More evidence points towards pubic hair trimming among women in an effort to mimic patterns seen in pornographic materials [16,17]. This may reflect a general cultural trend or a tendency to internalize the pubic hair patterns seen in pornography. One major psychological trend that has been studied is the increasing belief that extra hair makes patients feel ‘less feminine’, thereby leading to extra removal behaviors [24,25]. Additionally, there is a risk of genitourinary laceration during depilation, which is predominantly done by razors [26]. Adult females’ tendency to undergo labiaplasty procedures after exposure to pornography is still unclear as there is insufficient evidence in studies analyzed thus far [17]. Schick et al. noted a decreased likelihood to attempt risky sexual behaviors among women who were dissatisfied with their genital appearance, which may be one of the few protective instances against genital injury from perilous sexual maneuvers [15].

In a case series of six women with persistent genital arousal disorder, an association with certain dermatoses existed [14]. This was often accompanied by masturbation to relieve sensations of genital engorgement, often requiring the use of pornographic material. Further, after viewing media including pornography, adult females were more likely to have self-image issues, leading to pubic hair trimming patterns. However, any association between labiaplasty surgeries and masturbation remains unclear [15-17].

Risk of early sexual activity and mirroring acts of pornographic performers by children

Notable reports of masturbation-induced genitocutaneous injuries among adolescents focused on males. The first of two notable cases involved a urethrocutaneous fistula from the insertion of magnets into the urethra [18]. These magnets were initially inserted one year prior to the emergency department presentation. The other case involved salicylate toxicity secondary to the use of topical methylsalicylate as a masturbation lubricant [19]. In both cases, the use of pornography was not explicitly stated. Further investigation is needed to determine whether such cases arose due to material viewed by the adolescents.

As Wong et al. demonstrated, an increased likelihood of early sexual activity in adolescents who regularly consumed pornography exists [20]. Although no overt cutaneous manifestations were described, another cross-sectional study by Ng et al. in a Singaporean sexual health clinic found an increased frequency of encounters with sex workers among adolescents aged 16-19 years [21]. Many patients presented with cutaneous manifestations such as genital warts, ulcers, and molluscum.

In adolescents, recurring themes include attempting to recreate what is seen in pornographic materials and engaging in early sexual activity; either with peers or with sex workers [20,21]. These can have overt cutaneous manifestations, such as genital injury or lesions from sexually transmitted diseases. In a cross-sectional study of Swedish high school students, it was found that males with a high level of pornography consumption were statistically more likely to attempt acts seen in pornographic films, which can be problematic if they regularly view violent genres of pornography with risk of genitocutaneous injury [24].

Preemptive Management by Pediatricians

Children and adolescents have been found to be at risk for autoerotic asphyxiation, especially if there is a history of emotional dysregulation and sadomasochistic relationships. Choking games among the young with related cutaneous findings like strangulation marks and neck erythema may be early manifestations of autoerotic asphyxiation, as a subset of these progress to fatality [27]. Pediatricians should therefore be alert to such behavior to interrupt it and prevent further its development. 

Potential treatments and therapies

The lack of specific diagnostic criteria is also coupled with a lack of evidence-based management for pornography addiction. In one notable case series, a majority of male patients (17/19; 89%) with compulsive sexual behavior experienced a reduction in symptoms when taking naltrexone, with treatment ranging from 2-27 months. However, these patients were concurrently taking other psychotropic medications during naltrexone initiation [28]. 

In another case, a patient with obesity and cue-triggered snacking was prescribed topiramate 50 mg daily for weight loss. Coincidentally, he also noticed an improvement in trigger-associated compulsive sexual behaviors such as the consumption of prostitution. Both binge eating and consumption of prostitution reoccurred after the drug was discontinued, and decreased again after reinitiation [29]. Further research into these medications may be a first step into the potential pharmacologic management of pornography addiction and by extension, cutaneous injury. 

Risks among pornographic performers

Finally, among pornographic performers themselves, there is a high risk of cutaneous sexual disease transmissions such as herpes simplex and human papillomavirus as these are ubiquitous and not routinely monitored [30]. Instead, most sexually transmitted disease panels for performers are limited to HIV, gonorrhea, chlamydia, and syphilis. Pornographic films featuring unprotected sexual intercourse may encourage viewers to engage in unprotected sex without thought of the associated sexually transmitted infection and pregnancy risks. 

**Figure 2:**
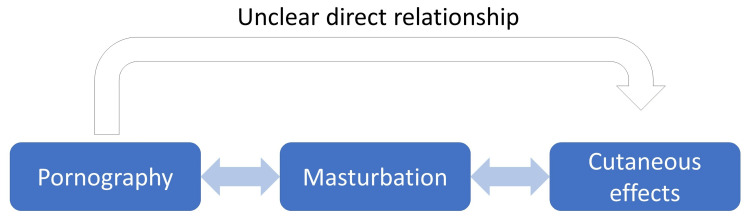
Current gap in literature pertaining to the direct link between pornography and cutaneous injuries or effects. Among studies evaluated, a link between pornography and sex acts like masturbation was studied extensively in adolescents [20,21,24,27]. Additionally, the rare cutaneous sequelae of excessive masturbation like hemorrhagic bullae, Fournier's gangrene, and penile asphyxiation were demonstrated in notable cases [8,10,13]. However, the direct link between pornography and cutaneous effects on genital and extragenital skin was not clearly evaluated in any of the studies included, thereby highlighting a notable gap for further study.

Overall, there may not be a clear picture of cutaneous manifestations of pornography addiction (Figure 2). Certainly, excessive masturbation can lead to genital injury, but the strength of their association still remains to be seen and is an area of further research. 

## Conclusions

There is no unified psychiatric classification of pornography addiction, which often falls into either internet addiction or hypersexuality disorders. Similarly, there is no consensus on the treatment of behaviors where excessive pornography consumption causes significant impairment in daily functioning. Our review of the literature yielded articles evaluating an association between pornography and masturbation, or masturbation and cutaneous injuries, with an unclear association between pornography and cutaneous pathologies.

Different behavioral patterns occur with cutaneous manifestations, often in the presence of pornography. Adult males are at risk of engaging in sexual behaviors with a risk of injury or death, like autoerotic asphyxiation. Adult females often trim their pubic hair to mirror pornographic performers. Children and adolescents are likely to replicate sexual acts seen in pornographic material, leading to an increased risk of early sexual activity, sex worker solicitation, and sexually transmitted diseases. Further research is needed to evaluate the direct relationship between pornographic addiction and genitocutaneous pathology with or without psychosocial changes. Relationships exist in the link between masturbation and skin injury, although the strength of association remains unclear and overt injury is quite rare. These major gaps in knowledge represent avenues for more thorough research.

## Appendices

Appendix 1

**Table 2:** Studies evaluated

Author	Study Category		Sample size	Study findings	Pornographic associations		Genitocutaneous findings	Treatments
Fong et al. 2021 [5]	Systematic review		N/A	Behavioral addictions can present in a variety of subtle and deceptive patterns. Because of the intense shame, guilt, and embarrassment felt by patients, it may fall to providers to utilize screening tools and deeper interviewing techniques to uncover the extent of these behaviors. Identifying when the line is crossed from recreation/habit to psychopathology relies on understanding current diagnostic criteria and consideration of cultural, ethnic, and local community standards. Individuals are also likely to cross back and forth between this line of pathology and habit, further clouding provider’s opinions of diagnosis; therefore, tracking and monitoring these symptoms over time is critical to establishing patterns of use and documenting ongoing consequences. Treatment for these conditions is emerging slowly, and treatment outcomes for these conditions appear to be similar to those with other addictive disorders.	Pornography has been found to manifest similar to other behavioral addictions.		No overt findings.	Cognitive behavioral therapy has shown some therapeutic benefit.
Slutske et al. 2013 [6]	Prospective cohort		4,764	Disordered gambling (DG) will soon be included along with the substance use disorders in a revised diagnostic category of the Diagnostic and Statistical Manual of Mental Disorders DSM-5 called ‘Substance Use and Addictive Disorders’. This was premised in part on the common etiologies of DG and the substance use disorders. Using data from the national community-based Australian Twin Registry, we used biometric model fitting to examine the extent to which the genetic liabilities for DG and alcohol use disorder (AUD) were shared, and whether this differed for men and women. The effect of using categorical versus dimensional DG and AUD phenotypes was explored, as was the effect of using diagnoses based on the DSM-IV and the proposed DSM-5 diagnostic criteria. The genetic correlations between DG and AUD ranged from 0.29 to 0.44. There was a significantly larger genetic correlation between DG and AUD among men than women when using dimensional phenotypes. Overall, about one-half to two-thirds of the association between DG and AUD was due to a shared genetic vulnerability. This study represents one of the few empirical demonstrations of an overlap in the genetic risk for DG and another substance-related addictive disorder. More research is needed on the genetic overlap between DG and other substance use disorders, as well as the genetic overlap between DG and other (non-substance-related) psychiatric disorders.	No explicit associations		No explicit genitocutaneous findings	No treatments described but strong genetic component to addictive behaviors.
Verma et al. 2020 [8]	Case Report		23-year-old male presented with a painless blister following masturbation the same morning.	Hemorrhagic bulla superimposed on penile lichen planus.	Unclear. Not explicitly mentioned.		Localized to glans and prepuce only.	Treated with aspiration and topical 0.1% tacrolimus
Ghiya et al. 2008 [9]	Case report		30-year-old male presented with a 3-month history recurrent itching, swelling, and redness a few minutes after masturbation that persisted for 4-6 hours.	Masturbation-induced urticaria. Symptoms did not occur during protected or unprotected vaginal intercourse. No other history of dermographism or urticaria.	Unclear. Not explicitly mentioned.		Localized to penis.	Treated with hydroxyzine 10 mg and prednisolone 20 mg for 5 days which prevented further urticaria.
Heiner et al. 2012 [10]	Case report		29-year-old male with a history of masturbation-induced penile erythema presented with a two-day history of fever, severe scrotal pain, and diffuse myalgias. He endorsed frequent masturbation using soap as a lubricant.	Significant erythema, edema, and warmth of area including penis (but sparing glans), scrotum, and pubic symphysis. Large eschar noted on ventral penis. Fournier’s gangrene diagnosed.	Unclear. Not explicitly mentioned.		No gangrenous extension to bladder or rectum. Patient suffered from septic shock with a complicated hospital course.	Multiple debridement surgeries, aggressive antibiotic treatment, and split-thickness skin grafting.
Focardi et al. 2018 [11]	Case series		Report of three cases. Case 1, 52-year-old man partially hung by neck and ankles to ceiling. Case 2, 31-year-old man found on bed with canisters of butane gas. Case 3, 52-year-old man found on couch with multiple medications (including sildenafil and diazepam) and used syringes.	Accidental death in autoerotic maneuvers (AAD). Case 1, soft furrow of skin in neck demonstrated likely cardio-respiratory arrest secondary to mechanical asphyxia. Case 2, numerous petechiae and purpura. Case 3, puncture wounds.	Pornographic materials (pictures, magazines) found in each case. Active masturbation near the time of death was occurring in Case 2 and Case 3.		Case 2: poly-visceral congestion likely due to acute asphyxia.	All deaths were ruled accidental. None of the patients had prior psychiatric diagnoses. Epidemiological data shows high prevalence of men in AAD.
Vennemann et al. 2006 [12]	Case report		30-year-old man found hanged in living room. Accidental death in autoerotic maneuver. Left hand was in trousers in genital region.	Strangulation marks on bilateral neck. Also had findings of advanced sarcoidosis, including nasal granulomas.	Violent pornographic content found on computer which was likely being viewed close to time of death.		Iridocyclitis, uveitis, and diffuse granulomas in bilateral lungs, spleen, and abdominal lymph nodes.	Suicide could have been provoked by advanced sarcoidosis, which had poor prognosis due to nasal granulomas and chronic uveitis.
Kyomukama et al. 2021 [13]	Case report		43-year-old male presented with a 72-hour-history of a ball bearing wrapped around penile shaft.	Ulcerated areas on penis, grossly edematous penile shaft with serosanguinous discharge.	Patient had a history of pornography addiction.		Primarily genitourinary involvement.	Surgical debridement and broad spectrum antibiotics.
Adult Females								
Goldmeier et al. 2008 [14]	Case series	Report of six cases of women with persistent genital arousal disorder and evaluation of different parameters of the disorder including cutaneous, psychosocial, and physiological.		Persistent genital arousal disorder coexisted with dermatoses in certain cases.	Unclear. Not explicitly mentioned. Masturbation is often used as a method to reduce feelings of genital arousal.	Primarily engorgement of genitalia.		Associated with neurophysiological conditions such as anxiety and pathologic conditions like genitopelvic prolapse.
Schick et al. 2010 [15]	Cross-sectional study	217 undergraduate women surveyed for satisfaction of genital appearance and sexual activity.		General dissatisfaction with genital appearance (labia majora, labia minora, pubic hair, etc.) was associated with decreased sexual satisfaction and decreased likelihood of pursuing risky sexual behaviors that could be injurious to genitalia.	Increased exposure to pornography may increase feelings of distress about genital appearance.	Self-esteem and self-consciousness issues primarily.		Sample was primarily Caucasian, heterosexualcollege-aged women.
Dubinskaya et al. 2022 [16]	Cross-sectional study	25 most-viewed videos from popular pornography sites were reviewed. Female genital appearance, hair grooming, pigmentation, and labial dimensions were assessed.		Among male and female performers, complete pubic hair shaving was the most common pattern, followed by trimming. 8% of videos showed surgically enhanced labia majora. Most videos had a range of genital appearance, weakening association with labiaplasty, but reinforcing a connection with hair grooming patterns.	Explicitly pornographic videos from the five most popular free pornography websites.	No other pelvic organ prolapse signs, piercings, or extracutaneous findings noted.		
Mowat et al. 2015 [17]	Systematic review	Online pornographic content and female attitudes towards genital cosmetic surgery.		Vulvar diversity pathologized in most of cyberspace, “clean slit” vulva perceived as ideal.	Internet and pornography may have a role in women’s likelihood of undergoing labiaplasty surgery.	Primarily psychological manifestations including self-esteem.		Lack of definitive data on association between pornography and labiaplasty.
Children/Adolescents								
Kinjo et al. 2019 [18]	Case report	14-year-old boy with fever, urinary incontinence, and scrotal pain. Physical examination showed induration at pubic symphysis.		Urethrocutaneous fistula and abscess secondary to spherical magnets inserted into urethra one year prior to facilitate masturbation.	Unclear. Not explicitly mentioned.	Fever, urethrocutaneous fistula.		According to a review of Japanese literature, among 51 cases of urethrovesical foreign body in patients under 18, masturbation was the cause of 31 (73.8%).
Thompson et al. 2016 [19]	Case report	14-year-old boy presented to the emergency department with dyspnea, chest pain, lightheadedness, and fatigue.		Salicylate toxicity. Used 60-gram tube of methylsalicylate cream as lubricant to masturbate. No overt cutaneous lesions.	Unclear. Not explicitly mentioned.	High anion gap metabolic acidosis.		Absorption was likely at scrotal skin, which has been shown to have a 40-fold greater absorption of certain compounds compared to other dermal areas.
Wong et al. 2009 [20]	Case-control	500 Singaporean adolescents assessed for sexual activity predictors.		No overt cutaneous manifestations described.	Among boys, risk factors for early sexual activity included pornography exposure [OR]: 5.82 [95% confidence interval [CI]: 2.34-14.43].			
Ng et al. 2016 [21]	Cross-sectional	300 male adolescents aged 16-19 at an STD clinic in Singapore, assessing for predictors of sexual activity.		Diagnosed STI’s after sexual encounters with female sex workers. These include genital herpes, genital warts, molluscum contagiosum, infectious syphilis, pubic lice, and HIV.	Increased frequency of pornography viewing was associated with more encounters with female sex worker (adjusted prevalence ratio 1.47, CI: 1.04-2.09).	STI involvement including urethritis, pharyngitis, proctitis		
Behrendt et al. (2002)	Case series	4		Deaths of 4 young women from autoerotic asphyxiation in the presence of erotic paraphernalia	Pornographic materials were found in each case, many depicting violent sex acts.	Bruises, strangulation marks, and lacerations found.		
Sauvageau et al. 2006	Review of case reports	408		Predominantly caucasian males with a preponderance of erotic material found in autoerotic deaths described in literature.	Pornographic material was found in most cases of autoerotic asphyxiation.	Many cases involved foreign body insertion and electrocution which left signs such as burns and lacerations.		
Häggström et al. 2005	Prospective cohort	718		Pornography consumption and sexual behaviour were studied, with an aim to investigate any associations. Participants were 718 students from 47 high school classes, mean age 18 years, in a medium-sized Swedish city. More men (98%) than women (72%) had ever consumed pornography. More male high consumers than low consumers or women got sexually aroused by, fantasized about, or tried to perform acts seen in a pornographic film (P<0.001). Three-quarters of the sample had had sexual intercourse, of which 71% reported contraceptive use at first intercourse. Anal intercourse was reported by 16%, with infrequent condom use (39%). Intercourse with a friend (adjusted odds ratio (adj. OR) 2.29; 95% confidence interval (CI) 1.27-4.12) was significantly associated with high consumption of pornography among men, while anal intercourse (adj. OR 1.99; 95% CI 0.95-4.16) and group sex (adj. OR 1.95; 95% CI 0.70-5.47) tended to be associated. A significant confounder was early age of sexual debut (adj. OR 1.49; 95% CI 1.18-1.88).	Increased pornography viewing was associated with increased high risk sexual behaviors among adolescents.	STDs like genital herpes and molluscum common. Students tried to replicate extreme or injurious sexual scenes viewed in pornography.		Some form of preemptive therapy and outreach was discussed but no elaboration.
Verinis et al. 1970	Cross-sectional	60		Among male and female undergraduate students, increased body hair was seen as more virile and less feminine.	No explicit associations	Female students were more likely to remove pubic hair at the conclusion of the study.		
Glass et al. 2012	Cross-sectional	335		Objective: To describe the demographics and mechanism of genitourinary (GU) injuries related to pubic hair grooming in patients who present to U.S. emergency departments (EDs). Materials and methods: The National Electronic Injury Surveillance System contains prospectively collected data from patients who present to EDs with consumer product-related injuries. The National Electronic Injury Surveillance System is a stratified probability sample, validated to provide national estimates of all patients who present to U.S. EDs with an injury. We reviewed the National Electronic Injury Surveillance System to identify incidents of GU injury related to pubic hair grooming for 2002-2010. The variables reviewed included age, race, gender, injury type, location (organ) of injury, hospital disposition, and grooming product. Results: From 2002 to 2010, an observed 335 actual ED visits for GU injury related to grooming products provided an estimated 11,704 incidents (95% confidence interval 8430-15,004). The number of incidents increased fivefold during that period, amounting to an estimated increase of 247 incidents annually (95% confidence interval 110-384, P = .001). Of the cohort, 56.7% were women. The mean age was 30.8 years (95% confidence interval 28.8-32.9). Shaving razors were implicated in 83% of the injuries. Laceration was the most common type of injury (36.6%). The most common site of injury was the external female genitalia (36.0%). Most injuries (97.3%) were treated within the ED, with subsequent patient discharge. Conclusion: Most GU injuries that result from the use of grooming products are minor and involve the use of razors. The demographics of patients with GU injuries from grooming products largely paralleled observations about cultural grooming trends in the United States.	No explicit associations	Most injuries involved a shaving razor and included lacerations, burns, and erosions. Female genitalia were more likely to be injured,		Most injuries successfully treated in ED and discharged.
Cowell 2009	Retrospective cohort	N/A		Objective: Voluntary asphyxiation among children, preteens, and adolescents by hanging or other means of inducing hypoxia/anoxia to enhance sexual excitement is not uncommon and can lead to unintended death. This study addresses autoerotic asphyxiation (AEA) with the intent of increasing pediatricians' knowledge of the syndrome and awareness of its typical onset among young patients. AEA is characteristically a clandestine and elusive practice. Provided with relevant information, pediatricians can identify the syndrome, demonstrate a willingness to discuss concerns about it, ameliorate distress, and possibly prevent a tragedy. Methods: A retrospective study was undertaken of published cases both fatal and nonfatal and included personal communications, referenced citations, clinical experience, and theoretical formulations as to causation. Characteristic AEA manifestations, prevalence, age range, methods of inducing hypoxia/anoxia, and gender weighting are presented. All sources were used as a basis for additional considerations of etiology and possibilities for intervention. Results: AEA can be conceptualized as a personalized, ritualized, and symbolic biopsychosocial drama. It seems to be a reenactment of intense emotional feeling-states involving an identification and sadomasochistic relationship with a female figure. Inept AEA practitioners can miscalculate the peril of the situation that they have contrived and for numerous reasons lose their gamble with death. Conclusions: Pediatricians should be alert to the earliest manifestations of AEA. Awareness of choking games among the young and, of those, a subset who eventually progress to potentially fatal AEA is strongly encouraged among all primary care professionals who may be able to interrupt the behavior.	Pornographic material viewing was a way of early exposure to autoerotic asphyxiation.	Voluntary asphyxiation primarily led to strangulation marks around the neck area.		Pediatricians could intercept the early manifestations of autoerotic asphyxiation.
Raymond et al 2010	Case series	19		Background: Compulsive sexual behavior (CSB) is generally characterized by recurrent and intense sexually arousing fantasies, sexual urges, and behaviors, which cause individuals distress or impair daily functioning. Descriptive studies of individuals with paraphilic and nonparaphilic CSB indicate that they experience urges to engage in problematic sexual behavior. The opiate antagonist naltrexone has been successfully used to treat a number of disorders in which urges to engage in problematic behavior are a central feature, such as alcoholism. We hypothesized that naltrexone would reduce the urges and behaviors associated with CSB. Methods: Records of 19 male patients with CSB who were treated with naltrexone at an outpatient adult sexual health clinic were retrospectively reviewed. Results: Nearly all patients were already taking other psychotropic medications when naltrexone was initiated. Seventeen (89%) of the 19 patients reported a reduction in CSB symptoms when taking naltrexone for a period ranging from 2 months to 2.3 years, as judged by Clinical Global Impression scores of 1 or 2, indicating "very much improved" or "much improved." Five (26%) of the 19 patients chose to discontinue the medication. Conclusions: Naltrexone may be a useful adjunctive treatment for CSB.	Patients treated with naltrexone had reduced pornographic addiction impulses	No explicit genitocutaneous findings		Naltrexone could be an adjunctive treatment for compulsive sexual behavior
Khazaal 2006	Case report	1		Background: Among the multiple mechanisms of action of topiramate, AMPA/kainate antagonism may be particularly interesting for the treatment of disorders characterized by conditioned cognitive and behavioral cue reactivity. Case presentation: We report the case of a patient consulting primarily for obesity and cue triggered snacking, who responded well on topiramate at doses up to 50 mg. Coincidentally he reported on an improvement of compulsive nonparaphilic sexual behaviors (consumption of prostitution), which was also strongly triggered by environmental cues. Both addictive behaviors (snacking and consumption of prostitution) reoccurred after discontinuation of topiramate and again responded reintroduction of the drug. Conclusion: The present case report of topiramate's effect on comorbid obesity and nonparaphilic addiction could be interpreted as a further indication that topiramate acts on the common pathway underlying conditioned behaviors and seems to be a treatment of behavioral disorders associated with environmental cues.	Prostitution consumption and pornographic addiction reduced with topiramate.	No explicit genitocutaneous findings		Topiramate could have a combined effect on obesity and nonparaphilic addiction.
Kluger 2014	Systematic review			High rate of sexually transmitted illnesses in the adult film industry including genital herpes, HPV, and molluscum	Pornographic film performers	Condyloma, genital sores		
